# Magnetic resonance brain imaging in patients with visual vertigo

**DOI:** 10.1002/brb3.402

**Published:** 2015-10-14

**Authors:** Lea Pollak, Michael Osherov, Nadav Berkovitz, Inessa Beckerman, Rafael Stryjer, Sigal Tal

**Affiliations:** ^1^ Department of NeurologyThe Assaf Harofeh Medical CenterZerifinIsrael; ^2^ Affiliated to The Sackler Faculty of MedicineTel Aviv UniversityTel AvivIsrael; ^3^ Department of RadiologyThe Assaf Harofeh Medical CenterZerifinIsrael; ^4^ Public Health HospitalBeer YaacovIsrael

**Keywords:** Disability, MRI findings, visual vertigo

## Abstract

**Introduction:**

Patients with visual vertigo (VV) report dizziness provoked by moving visual surroundings. It has been suggested that these subjects develop a compensation strategy for a vestibulo‐proprioceptive deficit and rely excessively on visual input. We have postulated that patients with VV might have brain abnormalities that interfere with appropriate processing of visual stimulation and performed a brain MRI study to verify this hypothesis.

**Materials and Methods:**

Patients with VV of more than 3 months duration were included. They were asked to complete the Situational Characteristic Questionnaire (SCQ) that scores for the symptoms of VV. Dizzy patients without VV served as controls. A brain MRI was performed with a Siemens 1.5 Tesla scanner in patients and controls.

**Results:**

Twenty‐four patients with VV were included. Their mean SCQ score was 1.45 ± 0.9 (normal 0.16 ± 0.28). In 50% of patients, abnormalities in MRI imaging were found. Thirty‐three percent of 27 controls demonstrated an abnormal brain MRI. The two groups were similar in respect to the prevalence of a localized hemispheric or posterior fossa lesion (*P* = 0.13), but VV patients had more unspecific white matter brain changes than controls (*P* = 0.009). Patients and controls did not differ in age and gender distribution (*P* = 0.9) or the history of a neurotological event preceding their symptoms (*P* = 0.3).

**Conclusions:**

Our study suggests that multiple white matter lesions might contribute to occurrence of the phenomenon of VV. Future prospective large‐scale studies by specific MR techniques are indicated to validate our preliminary findings and elucidate the pathological mechanism of VV.

## Introduction

Some patients with dizziness are more susceptible to visual motion than others (Guerraz et al. 2001; Bronstein 2004, 2005; Bronstein et al. 2013). They suffer from discomfort, disequilibrium, and exacerbation of their dizziness when walking in supermarket aisles, crowds, while watching moving scenes, or looking at disco lights or moving strips. This phenomenon was called visual vertigo (VV). The cause of VV is unknown and is a matter of ongoing discussion in the literature.

It has been demonstrated that tilted or moving visual surroundings have more impact on perception of verticality and postural stability in patients with VV than on controls (Pavlou et al. 2006). Some individuals have a clear history of a vestibular insult preceding the onset of VV such as vestibular neuronitis or benign paroxysmal positional vertigo (BPPV). In others, no history of a vestibular event can be obtained, but abnormalities are found on vestibular testing. It has been suggested that these subjects develop a compensation strategy for their vestibulo‐proprioceptive deficit and rely excessively on the visual input rendered thus “visually dependent” (Bronstein 2004, 2005). Lastly, in a proportion of VV patients, no vestibular lesion can be detected and their symptoms have been often considered to be of psychological origin (Schniepp et al. 2014; Staab et al. 2014). However, most reports have shown that visual dependence is not related to an anxiety trait or a psychiatric disease. Since routine vestibular examination methods examine mainly the horizontal semicircular canal while testing of other parts of the peripheral vestibular system is available only at specialized centers (vestibular evoked myogenic potentials or video ENG), a vestibular lesion can remain undetected in these individuals. In addition to patients with peripheral vestibular dysfunction, patients with central vestibular diseases can also complain about VV, but visual dependence in patients with CNS has not been systematically studied.

We have postulated that patients with VV might have brain abnormalities that interfere with appropriate visual processing of abundant visual stimulation and report the brain magnetic resonance imaging (MRI) findings in patients with and without VV.

## Methods

Patients over the age of 18 who attended an outpatient dizziness clinic and complained about VV of more than 3 months duration were included in the study. Individuals with dizziness but no visual vertigo lasting longer than 3 month served as controls.

Visual vertigo was defined as a feeling of dizziness or discomfort caused by visual motion stimuli such as walking between supermarket aisles or in crowds, watching moving objects and scenes, traveling on escalators, or in a car on winding roads. Dizziness comprised complaints such as light headedness, giddiness, faintness, or subjective imbalance. We recorded the patients' medical history and present medication.

All patients had a detailed clinical neurotological examination, which included examination of the eye movements, head‐thrust test, head shaking test using Frenzel's glasses, examination of stance and gait, and positioning testing. The latter consisted of the Dix–Hallpike test and horizontal canal test. Where appropriate, a cardiovascular workup which included blood pressure monitoring, measuring of orthostatic hypotension, transthoracic echocardiogram, and 24–48 h Holter ECG was performed. Patients had also a standard audiogram and brainstem auditory evoked potentials. Excluded were patients with a previous neurological or psychiatric disease, patients with a cardiovascular condition causing dizziness (arrhythmia, valve problem or orthostatic hypotension) as well as patients where dizziness could be attributed to present medication (i.e., antiepileptic drugs, alpha1‐blockers or antihypertensives).

The patients were asked to complete the Situational Characteristic Questionnaire (SCQ). The questionnaire consists of nineteen questions scored from 0 (never) to 4 (always) that measure the frequency of symptoms provoked by environments with visual motion. The final score was obtained by dividing the total sum by the number of answered questions.

The MRI was performed with a Siemens 1.5 Tesla scanner (Siemens Aera). Directed protocol included: axial T2, sagittal T1, isotropic high‐resolution T2 3D (CISS) directed to pyramid of temporal bone, contrast injection, diffusion‐weighted imaging, axial FLAIR, axial and coronal T1 Fat Sat, T1 SPACE (isotropic 3D high resolution). Patients with cardiac, pacemaker, defibrillator, or metallic foreign body unstable in a magnetic field as well as patients with severe claustrophobia were excluded. Images were reported by an experienced neuroradiologist who was blinded to whether or not the patient suffers from visual vertigo. Small, punctuate lesions in the deep and periventricular white matter, hyperintense on all sequences were defined as white matter abnormalities (WMA).

### Ethical approval

The study was approved by the local Ethical Committee of the hospital and was conducted according to the principles of the Helsinki Declaration. All patients gave their signed consent.

### Statistical methods

We used the chi‐square test for comparing categorical variables. The *t*‐test was applied to compare means between variables with normal distribution. SPSS version 18 (SPSS Inc. Chicago, Il, 2008) was used for the analyses.

## Results

Twenty‐four patients with VV and 27 controls were recruited.

### Characteristics and MRI findings in patients with VV (Table 1):

**Table 1:** Characteristic and MRI findings in VV patients

Nr	Age	Sex	MRI findings	Medical history
1	59	F	WMA^a^	0
2	62	F	WMA	Mitral valve prolaps
3	44	F	Vascular loop VII, VIII lt	0
4	47	F	Colloid cyst of the 3rd ventricle	0
5	56	M	n	Ulcerative collitis
6	35	M	WMA	0
7	26	F	n	0
8	38	F	WMA	0
9	50	F	Left thalamic lacunar stroke	Thyroiditis
10	63	F	WMA	Sjögren syndrome
11	53	F	n	Hyperthyroidism, homocysteinemia
12	67	F	WMA	Ca breast
13	42	F	n	Fe def anemia
14	58	F	n	Diabetes mellitus, hypertension
15	28	M	n	0
16	72	F	WMA	Ulcerative collitis
17	48	F	n	Migraine
18	32	F	n	0
19	34	M	n	0
20	53	F	n	0
21	34	M	n	Idiopathic thrombocytopenic purpura
22	41	F	n	Hyperthyroidism
23	44	F	WMA	Mitral valve prolaps
24	60	M	WMA	Ischemic heart disease

a WMA, white matter abnormalities.

The mean age of VV patients was 47.7 ± 12.7 years, range 26‐72, six were males. Their mean SCQ score was 1.45 ± 0.9, range 0.2‐2.9. The most common trigger of visual vertigo was “looking at striped or moving surfaces such as curtains, venetian blinds, flowing water” (11%), “walking down a supermarket aisle” (9%), and “watching moving traffic or trains when trying to cross the street, or standing at the station”(9%).

In 11 patients (46%), the symptoms followed an acute neurotological event such as an attack of BPPV (eight individuals), labyrinthitis (one), whiplash injury (one), or recent onset of vestibular migraine (one). Two patients related the onset of symptoms to a stressful event while in the remainder (11) no reason for symptom onset was found.

The neurotological exam was normal in all patients at the time of presentation with VV.

In 12 (50%) of VV patients, abnormalities on MRI imaging were found. These consisted of nonspecific hemispheric white matter abnormalities (WMA) of less than 2 mm diameter (nine patients), a lacunar stroke in the basal ganglia (one patient), a colloid cyst of the 3rd ventricle without obstruction of the CSF flow (one patient), and a vascular loop of the cranial nerve complex VII–VIII (one patient).

### Characteristics and MRI findings in controls (Table 2):

**Table 2:** Characteristics and MRI findings in controls

No.	Age	Sex	MRI findings	Medical history
1	42	M	Lt frontal and lt cerebellar DVA	0
2	65	F	n	0
3	54	M	n	0
4	52	F	n	0
5	55	F	n	0
6	64	F	Lacunar stroke in basal ganglia	Epilepsy
7	43	F	Left hemispheric demyelinative lesion	0
8	47	F	n	0
9	41	M	Posterior fossa arachnoid cyst	Asthma bronchiale
10	28	F	n	0
11	55	F	n	0
12	57	M	n	0
13	36	F	n	Migraine
14	66	F	Lt VIII n enhancement	0
15	32	M	n	0
16	68	F	n	0
17	40	F	n	Migraine
18	38	F	n	0
19	49	F	n	0
20	56	F	n	0
21	47	M	WMA^a^	0
22	41	F	n	Systemic lymphoma
23	52	M	n	0
24	36	F	Posterior fossa arachnoid cyst	0
25	26	F	n	Obesity
26	64	F	n	Hypertension
27	44	M	WMA	0

a WMA, white matter abnormalities.

The mean age of controls was 48 ± 11.6 years, range 28–66, eight were males. The symptoms followed a preceding event in nine patients (33%): in three patients symptoms were triggered by trauma, in three the dizziness followed a BPPV cluster, and in further three, the symptoms were stress related. The neurotological examination was normal in all individuals.

MRI abnormalities were found in 9 (33%): two patients had WMA, and one had WMA and a lacunar stroke in the basal ganglia. In two patients, a posterior fossa arachnoid cyst without mass effect and in another a large hemispheric demyelinative lesion in the left hemisphere were detected. A further patient demonstrated a deep vein anomaly in the left frontal lobe and in the left cerebellum while a left acoustic nerve gadolinium enhancement without evidence of a vestibular schwannoma was found in another patient.

### Comparison between VV patients and controls (Table 3):

**Table 3:** MRI findings in VV patients versus controls

Types and localization of MRI abnormalities	VV (*n* = 24)	Controls (*n* = 27)	*P*
Number of patients with abnormal MRI	12	8	0.13
Hemispheric lesions			
WMA^a^	9	2	**0.009**
Demyelinative changes	0	1	0.3
Lacunar strokes in the basal ganglia	1	1	0.9
Frontal lobe deep vascular anomaly	0	1	0.3
3rd ventricle colloid cyst	1	0	0.2
Posterior fossa lesions			
Arachnoid cyst	0	2	0.1
Cerebellar deep vascular anomaly	0	1	0.3
VII and VIII th nerve abnormalities	1	1	0.9

a WMA, white matter abnormalities. Bold values are statistically significant.

Dizzy patients with VV were comparable with patients without VV in respect to age (*P* = 0.9), gender distribution (*P* = 0.7) and the history of a trigger (*P* = 0.3).

The frequency of abnormal MRI findings was detected at a similar rate in both groups of patient (Table 3). However, patients with VV had more unspecific white matter brain changes than patients without VV. Among nine VV patients with WMA, in three, the WMA were attributable to a vascular cause: in one patient to atherosclerosis, in another to hypercoagulability state, and in the third patient, the WMA were considered likely the result of Sjögren disease associated vasculopathy. None of the controls had a medical condition associated with the finding of WMA.

## Discussion

The presence of structural abnormalities on brain imaging in people with dizziness has been studied previously and the reports were controversial, possibly due to the small number of recruited individuals (Day et al. 1990). In one larger study, brain MRI findings of 125 dizzy individuals over the age of 65 were compared with those of 65 subjects without dizziness (Colledge et al. 2002). Structural abnormalities were common and consisted of cerebral atrophy, white matter lesions, and infarcts. Their presence did not differ between both groups except for midbrain white matter lesions that were more frequent in dizzy subjects. The authors concluded that routine MRI is unlikely to reveal a specific cause for dizziness.

This study was not aimed to reveal the cause of dizziness, but to search for possible structural brain changes that could explain the inappropriate visual dependence in patients with VV. We have found that dizzy patients with VV had no more structural abnormalities on MRI than dizzy patients without VV. The two groups were similar in respect to the prevalence of a localized hemispheric and posterior fossa lesion, or a vestibular nerve pathology. However, scattered white matter lesions were found in 37.5% of patients with VV, which was significantly more frequent than in patients without VV (7%) (*P* = 0.009).

The incidence of WMA in individuals without MS varies depending on MRI resolution and cohort's age (Katzman et al. 1999; Morris et al. 2009; Killiany 2010). WMA were noted in 31% of cases aged 40–49 years, and in 83% of those age 70 years and older. The lesions are nonspecific and may be seen with accelerated small‐vessel ischemic disease, vasculopathies, migraine, Lyme disease, and as a residual from inflammatory or traumatic brain damage (de Lau et al. 2009; Akashbi et al. 2012; Bashir et al. 2013). In 67% of VV patients in this study, the WMA were attributable to a vascular cause, while in three controls no underlying cause was identified. We assume that not the etiology of WMA but their multifocal distribution might account for the symptoms of VV.

Visual clue dependence can be a positive compensatory mechanism for vestibular‐ proprioceptive loss. It is the exaggerated response to visual stimuli that leads to VV. Vestibular stimulation leads to a low order sensory response in form of the vestibule–ocular reflex as well as to a high order sensory response in form of motion perception. The two reflexes can be uncoupled in certain situations such as in dancers who adapt to prolonged vestibular stimulation (Nigmatullina et al. 2013; Seemungal 2014). Vestibular perception has been shown to correlate with cerebral cortex white matter microstructure. The vestibular cortex white matter microstructure was found to be attenuated in individuals adapted to visual stimulation, but not in controls as demonstrated by diffusion‐tensor MRI imaging. This reflects the plasticity of the cerebral cortex and is in accordance with decreased vestibular cortical perception in adapted people, possibly as the result of decreased vestibular cortical stimulation possibly due to cerebellar gating (Nigmatullina et al. 2013).

Besides excitatory input from the vestibular organs, the vestibular cortex receives information from other systems such as the visual and auditory system. These stimuli are, in turn, inhibited by a feedback loop to provide an adequate vestibular perception. We hypothesize that in individuals who are visually dependent as the consequence of a peripheral vestibular lesion, the presence of multiple white matter changes interferes with subcortical areas that are responsible for feedback inhibition, leading, thus, to an exaggerated vestibular response to visual stimuli in form of visual vertigo.

Special attention has been paid to the presence of migraine in view of the knowledge that migraine can be associated with white matter lesions (Hougaard et al. 2014; Erdélyi‐Bótor et al. 2015). In our study, only one VV and 2 control patients – all with normal MRI findings – had a history of migraine. One limitation of this study is the relatively small number of patients. Another point to consider is the lack of localization and quantification of WMA by sophisticated voxel‐based analysis or Diffusion tensor imaging. Moreover, higher magnet strength imaging could possibly detect white matter changes also in individuals with VV whose neuroimaging has been reported to be normal.

The VV symptoms score in our cohort was similar to the values reported in the literature (Guerraz et al. 2001; Pavlou et al. 2006). Our controls denied any visual vertigo symptoms on direct questioning and were therefore not asked to complete the SCQ. Patients with vestibular dysfunction and no VV might positively answer some of the distinct queries of the questionnaire, as demonstrated previously (Guerraz et al. 2001; Pavlou et al. 2006). However, their mean SCQ score was significantly lower than in VV patients.

Recently, it has been demonstrated that depression is associated with deep and periventricular white matter hyperintensities on MRI (Wang et al. 2014; Birner et al. 2015). None of our patients had clinically significant depression or was treated by antidepressants. Since some authors consider VV to be of psychological origin, this issue should be addressed in future MRI studies of VV by also assessing anxiety and depression traits in respect to white matter changes.

### In conclusion

Our study suggests that multiple white matter lesions might contribute to the occurrence of the phenomenon of VV. Future prospective large‐scale studies by specific MR imaging are indicated to validate our preliminary findings and elucidate the pathomechanism of VV.

## Conflict of Interest

None declared.
